# Piezoelectric-Driven Amplification of Plasmon-Enhanced Fluorescence for Advanced Sensing Applications

**DOI:** 10.1021/acsami.5c03428

**Published:** 2025-05-05

**Authors:** Eni Kume, Ghadeer Almohammadi, Dominik Duleba, Aeshah Farhan M Alotaibi, Rongcheng Gan, Kseniia Mamaeva, A. Louise Bradley, Robert P. Johnson, James H. Rice

**Affiliations:** † School of Physics, University College Dublin, Belfield, Dublin 4, D04 V1W8, Ireland; ‡ School of Chemistry, University College Dublin, Belfield, Dublin 4, D04 V1W8, Ireland; § Chemistry Department, College of Science, University of Hafar Al Batin, Hafar Al-Batin, 31991, Saudi Arabia; ∥ Department of Physics, College of Science and Humanities, Shaqra University, Shaqra, 11961, Kingdom of Saudi Arabia; ⊥ School of Physics and AMBER, Trinity College Dublin, Dublin 2, D02 PN40, Ireland; # IPIC, Tyndall National Institute, Cork, T12 R5CP, Ireland

**Keywords:** plasmonics, fluorescence enhancement, piezoelectric polymers, detection, electric field

## Abstract

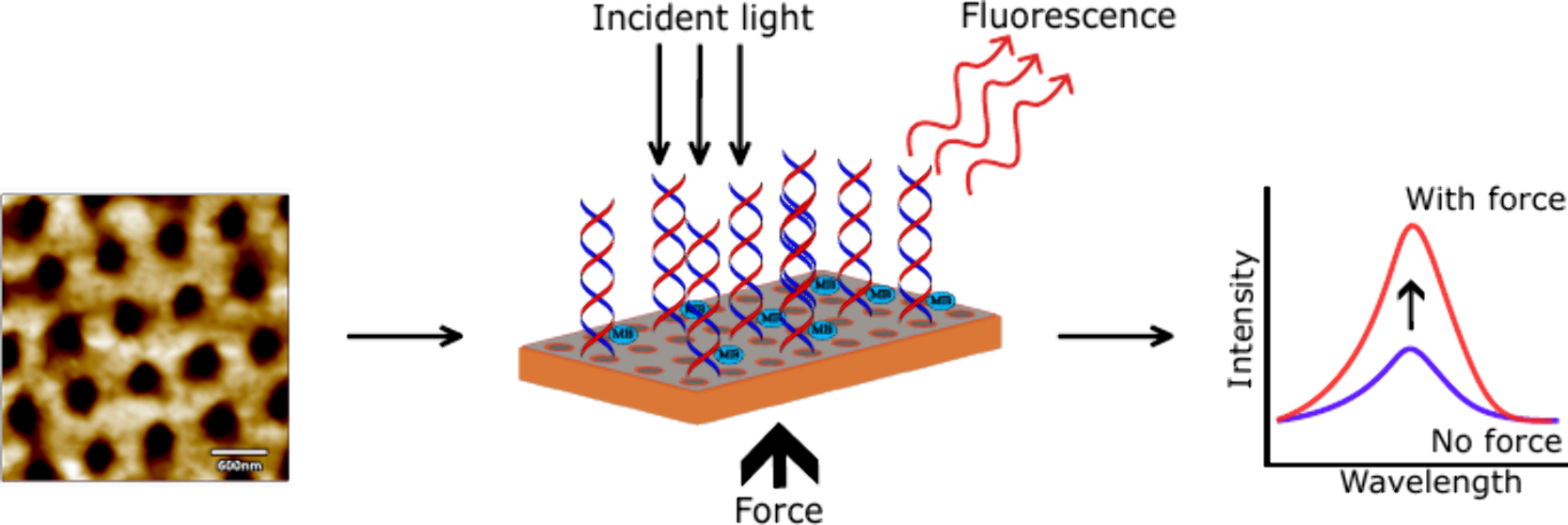

Fluorescence based detection is applied across various fields, including medical diagnostics and environmental sensing. A key challenge in these technologies lies in optimizing sensitivity through enhancement of the fluorescence signal. In this study, we demonstrate that combining piezoelectric and plasmonic processes increases the fluorescence yield. Piezoelectric poly(vinylidene fluoride-*co*-hexafluoropropylene) (PVDF-HFP), is utilized as an external electric field modulator to produce a reliable and reproducible fluorescence enhancement of InP/ZnS quantum dots approaching the single nanoparticle level. The relationship between the applied force and the fluorescence response is both experimentally quantified and theoretically modeled and the dependence of the fluorescence enhancement on the excitation wavelength and on the PVDF-HFP substrate topography is elucidated. Furthermore, fluorescence enhancement by a magnitude of order for a DNA hybridization assay on the gold-coated PVDF-HFP substrate is demonstrated, highlighting the practical applicability of this approach in biosensing.

## Introduction

Noble metallic nanostructures have become essential tools in modern bioanalytical technologies due to their unique plasmonic properties.^1^ These structures couple light to surface plasmon modes, enhancing optical signals near their surfaces. The approach known as plasmon-enhanced fluorescence (PEF), has advanced the sensitivity of fluorescence-based techniques by amplifying fluorophore brightness near periodic surfaces or nanoparticles.^1,2^

PEF is based on the overlap between the plasmon resonance of nanostructures and the absorption/emission spectra of fluorophores to boost signal intensity, thus leading to brighter and faster emission when they are located near the surface vicinity.^3,4^ The resulting increased excitation and emission rates of the fluorophores are related to the enhanced localized electromagnetic field of the surface.^2,5,6^ This enhanced electric field is a result of the excitation of surface plasmon polaritons, including propagating surface plasmon polaritons supported by films and waveguides, localized surface plasmons (LSPRs) supported by nanoparticles and surface lattice resonances in nanoscale periodic structures (gratings) as used in PEF.^4,7^ Fluorophores interact with the evanescent field and therefore the distance of the fluorophore from the surface plays a crucial role in the enhancement factor. If the fluorophore is too far, it will not be affected by the enhanced field, while if too close, quenching occurs due to nonradiative losses.^7^ Metal enhanced fluorescence (MEF) refers to the enhancement of fluorescence from the electromagnetic fields generated by metallic nanostructures, such as metal island films or metal nanoparticles and resulting in increased sensitivity of fluorescence-based detection methods.^5^ The most commonly used metals for plasmonic applications are noble metals like silver (Ag) and gold (Au) due to their biocompatibility and their high reflectivity, absorption, and scattering properties.^5,8^ At present, various systems have demonstrated a multifold increase in fluorescence signals, highlighting the promising potential of PEF and/or MEF for the development of innovative biosensors.^9^ Studies regarding plasmonic nanostructure design, such as anisotropic geometries, paper-based platforms, and flexible microfluidic chips, have expanded MEF’s versatility across different detection methods, including bulk fluorescence readouts and high-resolution imaging.^10,11^ These advances elucidate MEF’s versatility and its impact on sensing technologies.

Researchers have achieved notable fluorescence enhancement from the optimization of the spatial relationship between fluorophores and metallic surfaces, by improving plasmonic nanostructure design^8^ and optical enhancement mechanisms,^2^ while addressing challenges like signal quenching.^3^ This approach overcomes the limitations of conventional fluorescence-based techniques and enables new possibilities for biosensor development, based on nanostructured materials, improved fluorophore properties and plasmonic signal amplification,.^4,12^ These innovations are significant for applications ranging from disease diagnostics to environmental monitoring, and their integration in point-of-care testing (POCT) systems offers the opportunity to revolutionize clinical diagnostics by enabling cost-effective, portable, and highly sensitive devices.^4^

Fluorescence-based biosensors are key tools for molecular detection and analysis, offering rapid, selective, and highly sensitive readouts. However, these techniques confront challenges such as sensitivity limitations, signal instability, and interference from background noise, particularly in complex biological samples, despite significant progress.^13−15^ Strategies ranging from advanced signal amplification methods to novel material designs have sought to overcome these obstacles.^13−15^ For example, particle-based confinement on hydrogel arrays has accelerated DNA hybridization by two to 3 orders of magnitude via increased local concentration,^13^ while multiplexed biosensors combining Raman spectroscopy or lateral flow immunoassay with material engineering have achieved increased sensitivity.^14,15^ Using these insights, we developed a piezoelectric–plasmonic hybrid platform that utilizes the mechanical and dielectric properties of PVDF-HFP, alongside engineered nanostructures, to enhance fluorescence detection.

In this study, we researched a mechanically responsive fluorescence platform that integrates MEF-active nanostructures with a piezoelectric polymer substrate. We demonstrate how piezoelectricity^16−18^ can be utilized to further enhance plasmonic fluorescence yield by combining poly(vinylidene fluoride-*co*-hexafluoropropylene) (PVDF-HFP) with plasmonic metals. PVDF-HFP is a copolymer known for its chemical resistance, mechanical strength, thermal stability and high electrochemical stability, while displaying excellent piezoelectric properties.^19−21^ Its higher crystallinity and better mechanical properties in comparison to PVDF, enable the fabrication of rigorous PVDF-HFP films and membranes.^22^ These characteristics, combined with its nontoxic, safe and environmentally friendly nature, have sparked interest in various sectors, such as membrane technology,^23,24^ electrochemical energy storage^25^ and sensor technology for biomedical applications.^26−28^ Here, we demonstrate how the piezoelectric properties of nanoimprinted PVDF-HFP can be harnessed to further enhance the fluorescence amplification of metal nanostructures, serving as a complementary component to its intrinsic photonic and plasmonic characteristics.

In this work, the imprinted surface supports localized surface plasmon modes, while the piezoelectric substrate further modifies the local electric field in response to applied mechanical force. This interaction leads to the fluorescence output that can be controlled by adjusting the applied load, providing a controlled signal amplification. Our present study aligns with the broader MEF developments while offering a mechanically tunable means of achieving fluorescence enhancement. As a proof of concept, we utilized InP/ZnS quantum dots (QDs) to validate the ability of the hybrid material to enhance the detection sensitivity and demonstrate the applicability of the technology in hybridized DNA assay detection. Furthermore, we study the behavior of single quantum dots within this setup, examining how charge transfer, blinking, and fluorescence lifetimes are influenced by mechanical pressure. By uniting structural patterning, piezoelectric response, and photonic–plasmonic coupling, this platform establishes a versatile framework for enhanced fluorescence detection. Our findings highlight that incorporating a piezoelectric substrate significantly improves the sensitivity of both existing and prospective analytical methods and broadens the scope of possible bioanalytical and diagnostic applications.

## Results and Discussion

**Figure 1:**
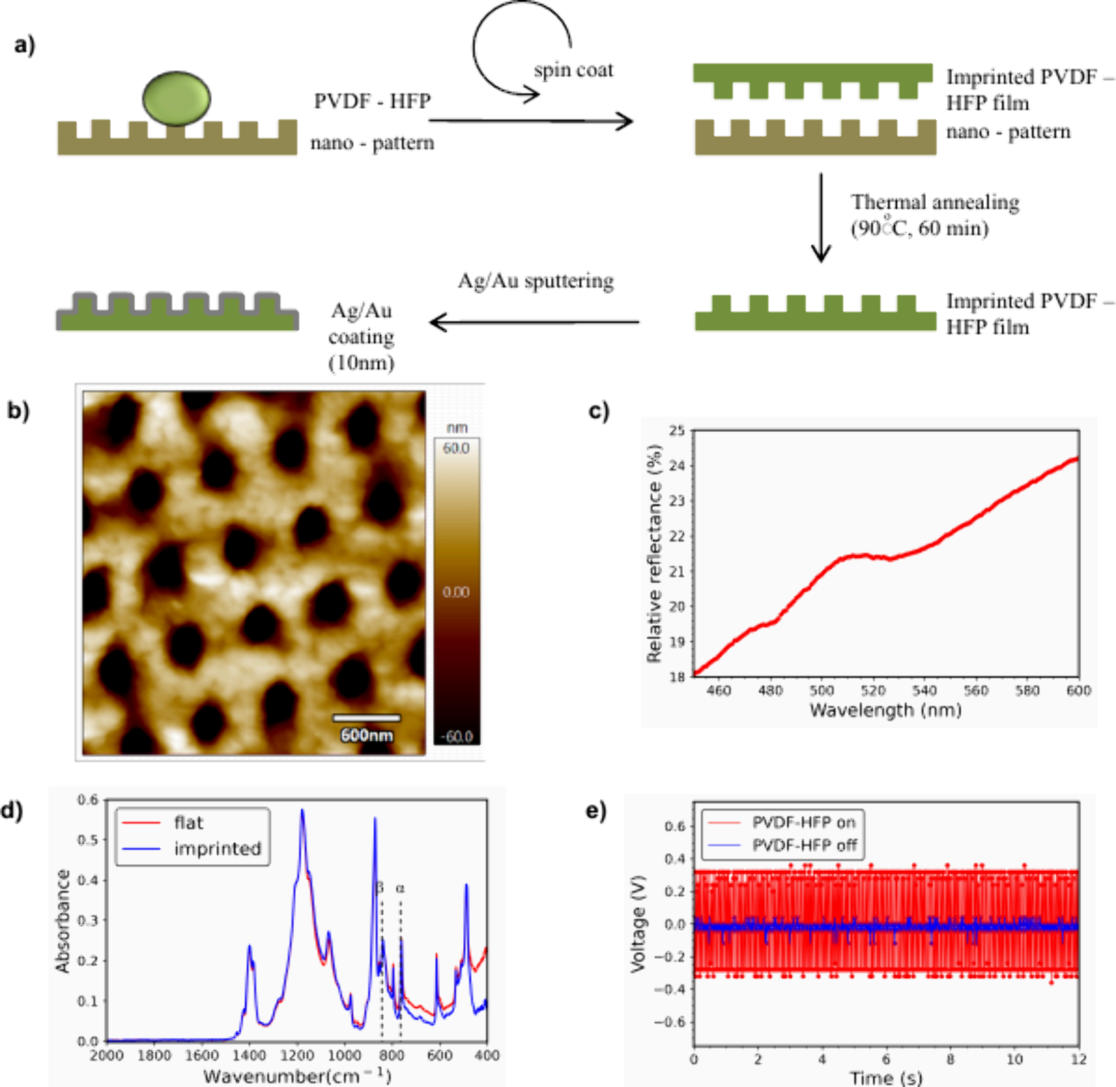
a) Simplified schematic of the fabrication process of imprinted PVDF-HFP thin films. b) AFM image of the imprinted pattern. c) Reflectance spectrum of a PVDF-HFP imprinted film. d) Infrared absorption spectrum (FTIR) of PVDF-HFP films used for the fluorescence spectroscopy study. e) PVDF-HFP film voltage response over time under sin wave external excitation.

The plasmon active metal (Ag or Au) coated imprinted PVDF-HFP films were first prepared and characterized as outlined in the Methods section. Briefly, to prepare the PVDF-HFP film imprinted with arrays of hole structures, a PVDF-HFP/DMF solution was placed on a silica nanopattern template. The film was then coated with 10/15 nm of a known plasmonic material (Ag or Au) using a standard evaporation process (Figure 1a). The imprinted film exhibits a periodic structure of circular holes with a period of approximately 700 nm. Atomic Force Microscopy (AFM) imaging images show an average distance between holes of 350 nm ± 15 nm, an average hole diameter of 350 nm ± 15 nm, and an average depth of 120 nm ± 30 nm (Figure 1b and Supporting Information S1). The coated imprinted surface having a period smaller than the excitation wavelength (473–633 nm) is expected to allow for the generation of additional localized and surface plasmon polaritons compared to coated flat films, leading to further enhancement of the fluorescence signal.^29^Figure 1c shows the specular reflectance of the fabricated samples. The Ag-coated imprinted sample shows small valleys in its reflectance, with local minima at 480, 530, and 700 nm, indicating the propagation of surface plasmons (plasmon resonances).^30^ In contrast, the flat samples do not exhibit any characteristic modes (Supporting Information Figure S3).

The piezoelectric activity of PVDF-HFP is related to the β-phase molecular arrangement of the polymer. In the β-phase, the molecular chains adopt a planar zigzag (all-trans) conformation, resulting in a highly polar structure. To assess the amount of β-phase in the nanoimprinted PVDF- HFP films, Fourier Transform Infrared Spectroscopy (FTIR) measurements were collected. Figure 1d shows the FTIR spectra of the imprinted and flat PVDF-HFP samples. The quantity of β-phase was calculated using the equation^31^
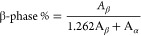
where *A*_α_ is the absorbance phase peak at 760 cm^–1^ and *A*_β_ is the absorbance phase peak at 840 cm ^–1^. The β-phase% of the fabricated samples was calculated at 43.35%–44.3%. To confirm the piezoelectric activity of the thin films, open-circuit voltage measurements were performed. The piezoelectric potential of the PVDF-HFP films is activated through the application of mechanical strain induced by a 20 g weight. This process generates a strong electrical potential, resulting in a measurable voltage (Figure 1e and Supporting Information Figure S5). These observations confirm that the piezoelectric potential can be activated by applying a mechanical weight.

### Piezoelectric-Assisted Plasmon-Enhanced Fluorescence

**Figure 2:**
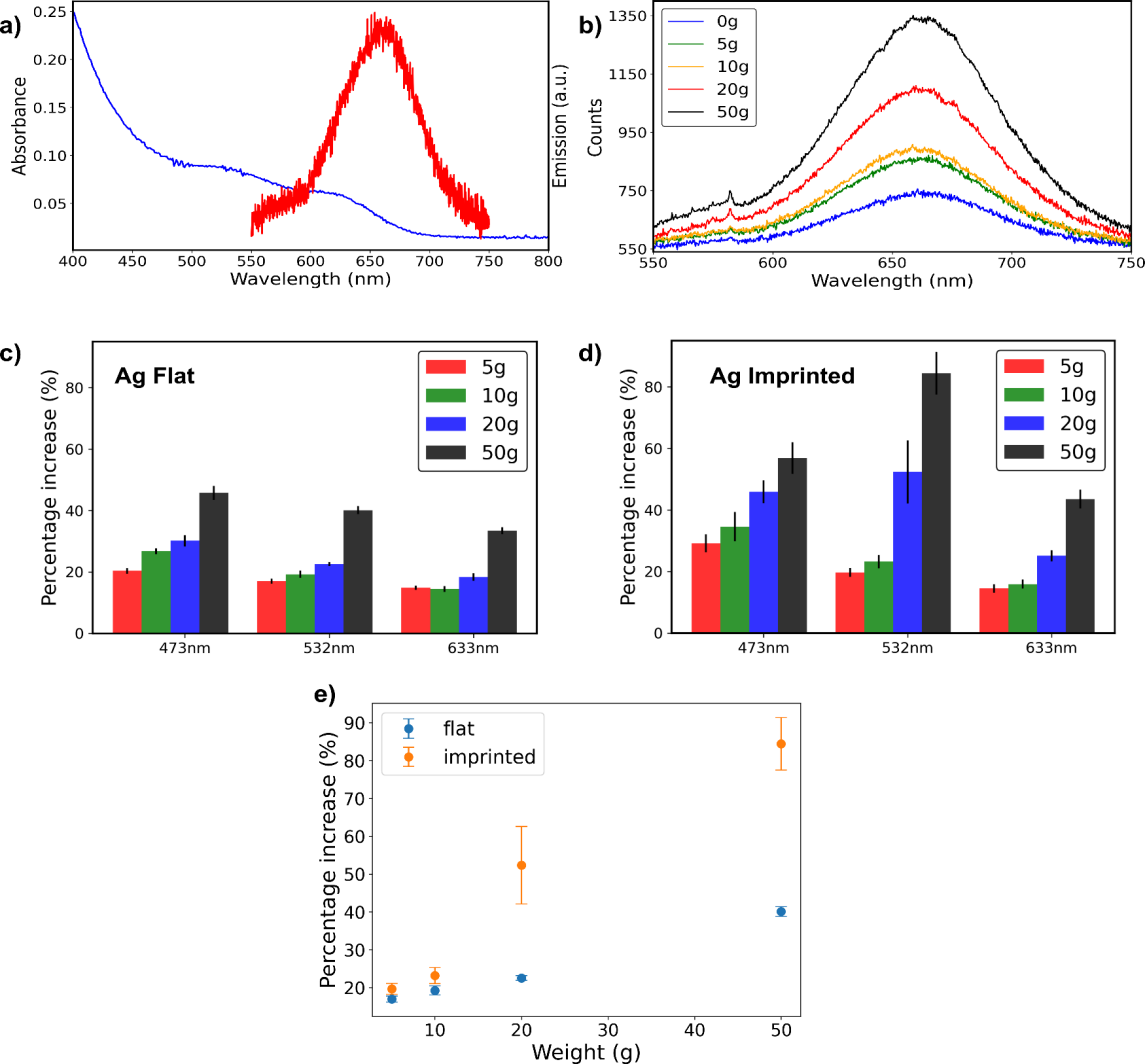
a) Absorption (blue line) and emission (red line) spectra of InP/ZnS quantum dots. b) Average emission spectra of InP/ZnS QDs in PMMA/toluene solution, spin-coated on a Ag-coated imprinted (hole features) PVDF-HFP film under different mechanical weights, using a 532 nm excitation wavelength. c–d) Fluorescence percentage enhancement (fluorescence intensity of the pressed state with weight vs nonpressed state) of InP/ZnS QDs on Ag-coated PVDF-HFP samples for various applied weights and excitation wavelengths. e) Scatter plot showing the fluorescence percentage increase of the tested samples under different weights for 532 nm excitation wavelength. Error bars represent the standard error of 9 measurements.

To evaluate the potential of metal-coated (Ag or Au) coated nanoimprinted PVDF-HFP to enhance fluorescence yield, studies were conducted using InP/ZnS QDs. The QDs (emission maxima at 660 nm, Figure 2a) were prepared in poly(methyl methacrylate) (PMMA) and spin-coated onto (Ag or Au) metal-coated nanoimprinted PVDF-HFP films. A second coverslip was placed over the QDs to enable mechanical deformation of the PVDF-HFP films when weight was applied. The InP/ZnS QDs exhibit an absorption maximum at 525 nm within the range of interest (450–700 nm), with a decreasing trend at higher wavelengths (Figure 2a).

Fluorescence measurements were performed for Ag-coated nanoimprinted PVDF-HFP with mechanical weights of various values (5 to 50 g) placed on top of the samples. The fluorescence intensity increased progressively with applied weight, reaching approximately twice the intensity at the highest weight (50 g) under 532 nm excitation (Figure 2b).

To further assess the capabilities of the metal-piezoelectric polymer thin film, a study was conducted using three different excitation lasers (473, 532, and 633 nm) in the visible wavelength range. The concentration of the QDs solution, the spin-coating and the experimental conditions were kept constant. The fluorescence signal percentage increase was calculated as
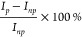
where *I*_*p*_ is the intensity of the pressed state and *I*_*np*_ is the intensity of the nonpressed state, where both imprinted and flat (nonimprinted) Ag-coated PVDF-HFP films were studied.

The impact of nanoimprinting the metal-piezoelectric thin films was examined. For Ag-coated flat PVDF-HFP films, an increase in fluorescence was observed for each wavelength, with a slight downward trend in fluorescence signal strength as the wavelength increased. The fluorescence enhancement under the heaviest weight (50 g) was 45% for 473 nm excitation, followed by a gradual decrease to 35% for 633 nm excitation (Figure 2c). Since the flat surface does not exhibit any plasmon modes that could couple with the incident light, the gradual decrease can be attributed to the increasing mismatch between the Ag surface plasmon extinction peak (located at 420 nm) and higher excitation wavelengths, as well as the lower absorbance of the QDs. In contrast, Ag-coated PVDF-HFP imprinted films exhibited significantly higher fluorescence enhancements: 60% for 473 nm excitation, 88% for 532 nm, and 45% for 633 nm excitation (Figure 2d). On average, the flat samples were half as effective as the imprinted ones (Figure 2e), indicating that the imprinted features contribute significantly to the enhancement of the fluorescence signal. Fluorescence measurements were collected from multiple spatial positions across the sample surface under each applied weight. Overall, the measured signal variation is 2% for the flat samples and about 5% for the imprinted ones, indicating that the load distribution across the film was sufficiently uniform for consistent fluorescence enhancement. Similar results were acquired for metal-piezoelectric thin films imprinted with a linear geometry (Supporting Information Figures S9 and S10).

The wavelength-dependent enhancement observed in imprinted samples is attributed to the coupling of excitation wavelengths with surface plasmon modes. In this system, coupled plasmons modes at 480 and 520 nm (as observed in the specular reflection spectrum, Figure 1c) provide optimal enhancement. Wavelengths that do not couple effectively with plasmon modes, such as 633 nm, exhibit lower fluorescence enhancement close to the flat surface value. The above results demonstrate that the integration of plasmonic, photonic, and piezoelectric properties can synergistically enhance spectroscopy systems, leading to a more refined and significant improvement in their performance.

The polymer concentration of PVDF-HFP was selected on the basis of reported literature, which ensures both film uniformity and favorable formation of the piezoelectric β-phase, although additional strategies (e.g., doping or altered processing steps) have been shown to further increase β-phase content.^32,33^ The metal thickness contribution was examined using simulations (Supporting Information Figure S7), elucidating the influence of the Ag/Au film thickness on the local electric field. These results show that the optimal thickness should be selected based on the specific analyte requirements (e.g., absorbance spectrum), to maximize the system’s fluorescence output. Although some of the parameters used in the present study may slightly deviate from the optimal ones, the fluorescence enhancement trends observed across different experimental conditions were reproducible and consistent with expected behavior. All the above suggest that the system operates as described, and that the enhancement mechanism is robust to minor deviations from ideal fabrication conditions. A detailed parameter sweep could be the subject of future work focused on device engineering and performance tuning.

### Mechanism for Fluorescence Enhancement

**Figure 3:**
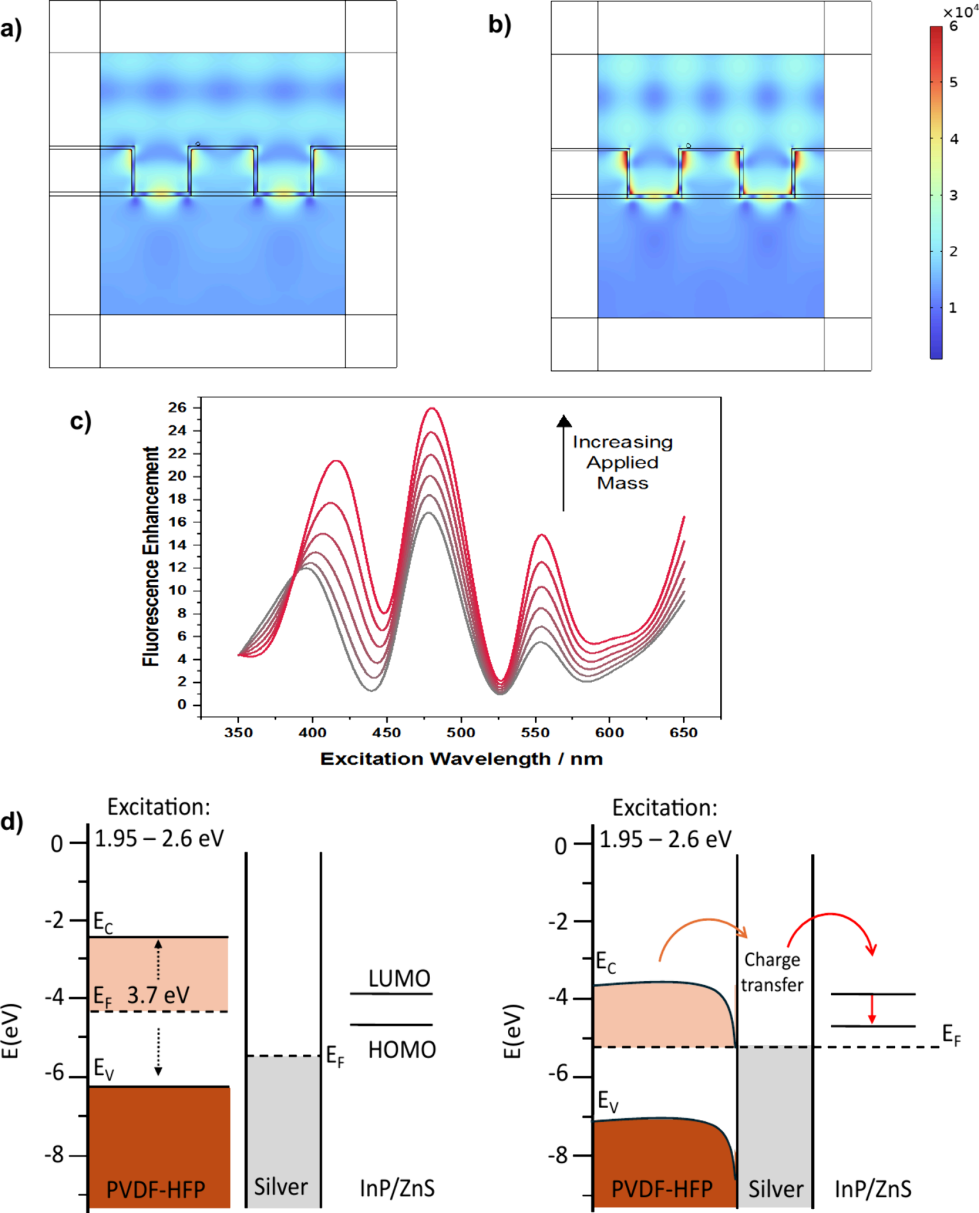
Finite element analysis studies of Ag-coated imprinted HPF-PVDF (a–c). a) Electric field enhancement around the nanotrench structure compared to a flat geometry before pressing using a 532 nm excitation wavelength. b) Electric field enhancement around the nanotrench structure compared to a flat geometry before pressing using a 532 nm excitation wavelength. c) Corresponding simulated fluorescence enhancement of InP/ZnS QDs on Ag-coated imprinted PVDF-HFP as a function of excitation wavelength, compared to a flat Ag-coated substrate. The gray region corresponds to no applied weight. d) Band diagram before (left) and after (right) contact between PVDF-HFP and Ag.

The mechanism of the observed increase in fluorescence signal intensity was investigated using finite element analysis. To simulate the fluorescence enhancement behavior of the imprinted piezoelectric polymer, finite element analysis via COMSOL Multiphysics was utilized. The system was represented as a 2D geometry of two periodic unit cells of the imprinted surface. The propagation of an incident beam perpendicular to the sample’s surface and polarized to the *x*-axis is considered, and the enhancement factor is calculated based on enhancements in the local electric field around the nanostructure, as well as by considering radiative and thermal dissipated powers.^34^ The influence of the applied load on the enhancement factor is qualitatively approximated by considering the application of surface current density on the surface of the piezoelectric polymer Simulations show (Figure 3a) that the imprinted sample exhibits a significant electric field enhancement compared to the flat Ag surface before applying the load. This enhancement is further amplified within the cavity when the polymer is subjected to mechanical pressure (Figure 3b). The strongest electric field enhancement occurs at the edges of the nanoholes, suggesting the formation of localized photonic cavities at each hole. This process further amplifies the electromagnetic field in the vicinity of the plasmonic surface.

Considering the effective quantum yield of the QDs based on the radiative and nonradiative losses (see *Methods*), the fluorescence enhancement factor for the imprinted geometry was calculated (Figure 3c). Peaks in the enhancement factor correspond to plasmon resonance wavelengths (gray color), as confirmed by reflectivity measurements (Figure 1c). Under applied weight, the enhancement factor increases across the visible spectrum, although the magnitude of the increase varies with wavelength. Among the excitation wavelengths tested, 532 nm exhibited the highest percentage increase, followed by 473 and 633 nm (Figure 3c and Supporting Information S8e), consistent with experimental observations, meaning that the piezoelectric enhancement is consistent with the photonic enhancement of the system. The enhancement resonance peaks arise from the scattering of incident light through the cavities nanostructures (Purcell effect) and are influenced by the geometric specifications of the system.^35,36^ The observed increase in fluorescence signal under load is attributed to the piezoelectric properties of the PVDF-HFP substrate. Upon contact, the PVDF-HFP film forms an ohmic contact with the plasmonic metal,^37^ facilitating charge transfer across the interface (Figure 3d). Mechanical pressure induces an electric charge in the substrate due to its piezoelectric nature,^38−41^ generating charge transfer at the polymer–Ag interface.^42^ This charge transfer modifies the charge density and distribution on the Ag surface,^43,44^ further enhancing the local electric field, as confirmed by both experiments and simulations. The imprinted surface features on PVDF-HFP film provide photonic enhancement due to its structural features, complemented by additional electromagnetic field amplification arising from its piezoelectric properties. These changes alter the system’s resonance characteristics, causing a shift in resonance peaks, as predicted in Figure 3c.

Gold, a common alternative to silver in biological applications, was also tested by replacing the Ag coating with a 10 nm Au layer. Flat Au-coated PVDF-HFP films exhibited weaker plasmonic enhancement than their Ag-coated counterparts, with increases ranging between 18–22% and no variation across excitation wavelengths (Supporting Information Figure S6c). For imprinted Au-coated samples, the percentage enhancement was comparable to that of flat films under 473 and 633 nm excitation but showed a notable increase of approximately 40% under 532 nm excitation (Supporting Information Figure S6d). This enhancement was approximately half that observed with Ag coatings, likely due to the higher heat losses associated with Au. The pronounced enhancement at 532 nm aligns with the surface plasmon resonance of the imprinted PVDF-HFP substrate, as confirmed by COMSOL simulations, which predict plasmon resonance near 550 nm (Supporting Information Figure S8c). Additionally, the systematic blue shift in the emission peak (Supporting Information Figure S6b) supports the hypothesis of coupling with gold’s plasmon resonance induced by the piezoelectric charges, in agreement with the simulations (Supporting Information Figure S8c).

### Piezoelectric PEF Studies at the Single QD Level

**Figure 4:**
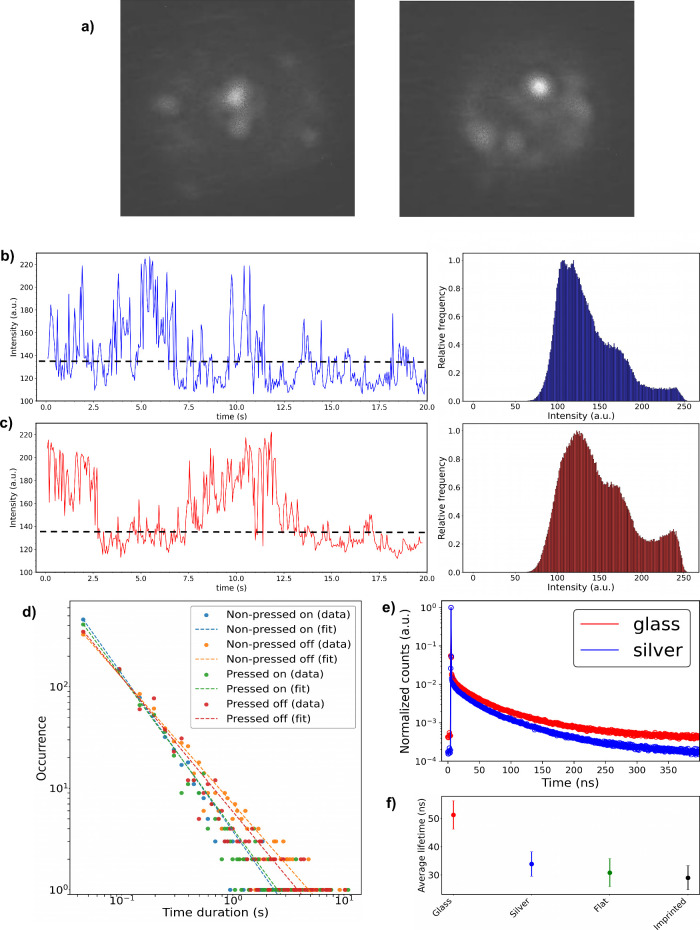
a) Instances of InP/ZnS QDs blinking. b) Time evolution of quantum dot intensity under no pressure (left graph) and the corresponding intensity histogram (right graph). c) Time evolution of quantum dot intensity under a 5g pressure (left graph) and the corresponding intensity histogram (right graph). d) Occurrence plot of on and off events for nonpressed and pressed samples, over the time duration of each event. e) Fluorescence decay curves of InP/ZnS QDs spin-coated on various substrates. Glass refers to a glass coverslip substrate and silver to Ag-coated substrate. f) Average lifetime of the QDs on each substrate with corresponding error bars.

To further investigate the underlying mechanisms of fluorescence amplification, the blinking behavior of QDs was analyzed before and after applying pressure (Figure 4a). Blinking refers to the stochastic switching of QDs between an emitting (fluorescence) on-state and a nonemitting off-state.^45^ This behavior provides critical insights into the physical and chemical properties of QDs, their surrounding environment, and the underlying mechanisms of their photophysics. During the on-state, photoluminescence arises from neutral QDs, while the off-state is associated with nonradiative Auger recombination or ionization of charged QDs.^45^ Additionally, a gray state exists when the radiative and Auger recombination rates are comparable.^46^ As shown in Figure 4b and 4c, all these states are evident in the quantum dots studied, likely due to their intrinsically low quantum yield (∼25%). For simplicity, gray states were categorized as on-states, with the threshold defined as *I*_*off_avg*_ + 1.1σ, where *I*_*off_avg*_ is the average intensity of off events. An average bin time of 50 ms was selected for the analysis.

The data reveals that mechanical pressure increases the percentage of on-states, corroborated by the blinking event occurrence plot (Figure 4d). For both pressed and nonpressed samples, the occurrences of “on” and “off” events follow a power-law distribution, *ct*^–*a*^, with deviations at longer time scales. For nonpressed samples, the power-law exponent *a* is 1.55 ± 0.01 for nonpressed on events and 1.24 ± 0.01 for off events. In contrast, for pressed samples, the value reduces to 1.49 ± 0.01 for on-events and increases to 1.32 ± 0.02 for off-events. These results show an increase in on events and a decrease in off events under mechanical pressure, suggesting a noticeable blinking suppression effect. Since the sample’s environment remains unchanged under load, the blinking suppression is likely attributed to an increased radiative rate for both neutral and charged QDs.^47^ This enhancement is driven by piezoelectric charge-induced energy states, resulting in an enhanced local electric field in agreement with the fluorescence spectroscopy measurements and simulation study.

Upon excitation, a fluorophore spontaneously emits photons through a radiative relaxation process characterized by its fluorescence lifetime. The fluorescence decay lifetime is not an intrinsic property of the fluorophore but is influenced by the surrounding environment. When a fluorophore is located near a metallic (plasmonic) surface, the enhanced electric field increases the radiative relaxation rate, typically leading to a shorter lifetime.^48^ This phenomenon is demonstrated in the present study of InP/ZnS QDs (Figure 4e). The average lifetime (τ_avg_) of QDs on a glass coverslip is 51 ± 5 ns, while on an Ag-coated glass coverslip, the lifetime is reduced to 34 ± 4 ns. In the presence of a nonpressed Ag-coated flat PVDF-HFP film, the lifetime is 31 ± 5 ns, and it is 29 ± 4 ns for a nonpressed Ag-coated imprinted PVDF-HFP sample (Figure 4f) as expected from a periodic nanocavity. On average, the presence of the Ag-coated PVDF-HFP film reduces the fluorescence decay lifetime, indicating strong interactions between the QDs and the Ag surface.

**Figure 5:**
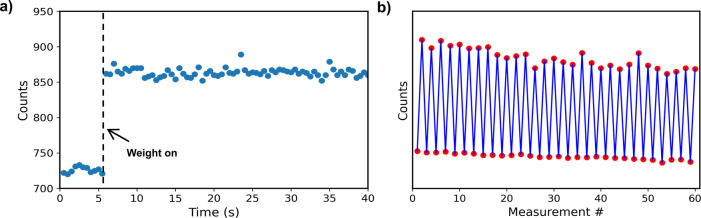
a) Sequential fluorescence measurements of InP/ZnS QDs on Ag-coated imprinted PVDF-HFP films were performed while adding/removing a 5 g weight. Each point represents the maximum intensity (in counts) of the fluorescence spectrum for a 0.5 s camera exposure time. b) Repeatability measurements, where each point represents the maximum fluorescence intensity after adding/removing a weight.

To study the stability of the system, time-resolved measurements were acquired. A 5 g weight was placed on the sample during fluorescence spectroscopy measurements. The acquisition time was set to 0.5 s, and the peak intensity of each fluorescence spectrum was identified and plotted over time (Figure 5a). After placing the weight, an immediate increase in intensity was observed within the 0.5 s of each data point. The intensity remained steady, within the margin of camera noise error, as long as the weight was kept in the same position. Upon removal of the weight, the fluorescence intensity returned to its original value (Supporting Information Figure S12). These measurements demonstrate the stability of the piezoelectric-induced enhancement. The piezoelectric properties of the PVDF-HFP, particularly the relationship between the electric displacement D and mechanical stress T: *D*_3_ = *d*_33_*T*_3_, where *d*_33_ is the piezoelectric constant^49,50^ - indicates that when the material is under pressure, an electric current is generated, and vice versa. The stored energy from the mechanical load leads to electric field generation, further amplifying the plasmonic response of the metal (Ag/Au). This effect shows excellent repeatability over multiple cycles (Figure 5b), with the fluorescence signal remaining stable over 30 repeated loading events and exhibiting less than 3% variation, indicating minimal degradation and strong mechanical durability, though some wear is expected after long-term repetitions.^51^

### DNA Hybridization Assay Studies

**Figure 6:**
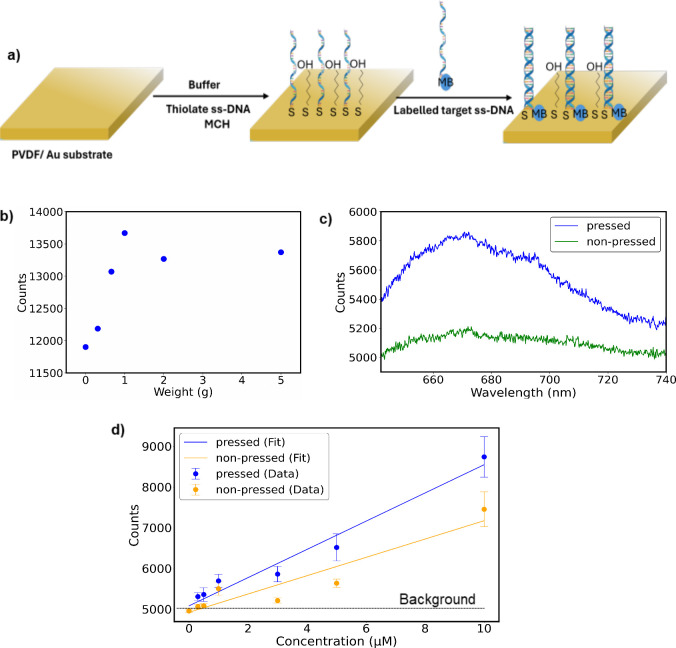
Studies using labeled DNA hybridization assay. a) Simplified schematic illustrating the fabrication process of the DNA hybridization assay. b) Fluorescence intensity dependence of 100 μM Methylene Blue (MB) on the applied mass. c) Fluorescence signal of MB labeled DNA assay imprinted sample (3 μMt) before and after applied load. d) Evolution of fluorescence intensity of MB at varying concentrations for nonpressed and pressed imprinted samples. Error bars represent the standard error of 8 measurements.

Next, we evaluated the applicability of the PVDF-HFP substrate in a DNA hybridization assay, which is widely used in biosensing for molecular detection.^52^ The PVDF-HFP substrate was coated with a 10 nm layer of Au, onto which probe ssDNA was immobilized. Mercaptocyclohexanol (MCH) was employed as a spacer to ensure the probe ssDNA was oriented perpendicular to the substrate surface.^53^ The target ssDNA, labeled with the fluorescent reporter Methylene Blue (MB), was introduced, and the sample was heated to promote hybridization (Figure 6a). To allow weight application during detection, a second coverslip was placed on top.

We investigated the effect of applied pressure. The sample remained stable under pressures up to 5 g, with the peak signal intensity observed at approximately 1 g (Figure 6b). The signal increased in the range of 0–1 g of applied pressure, attributed to the generation of piezoelectric charges. The percentage signal increase ranged from 14% to 11% for applied masses between 1 and 5 g, respectively. The relatively stable intensity is likely the result of two opposing effects: enhanced piezoelectric charges and the mechanical folding of DNA under pressure. At higher applied masses, the signal intensity dropped significantly, indicating that beyond a certain threshold, the hybridized DNA could no longer sustain the pressure, leading to structural deformation. Consequently, the target molecule (MB) approached the substrate, causing fluorescence quenching.

For fluorescence spectroscopy measurements, a 633 nm laser with a power output of 3 mW was employed as the excitation source. This wavelength was chosen to align with the absorption spectrum of MB, which has a peak at 664 nm (Supporting Information Figure S13). Since the excitation wavelength does not couple with the geometry of the imprinted sample, no significant fluorescence enhancement was anticipated in comparison to flat samples (Supporting Information Figure S6c and d). A complete study was conducted on the imprinted samples, which were expected to exhibit a slightly higher response compared to flat films. Fluorescence measurements were performed at 7–9 spots for each sample, and the average intensity was calculated for both the nonpressed and pressed states. All tested samples displayed an enhanced signal during pressing, with an increase of approximately 10% ± 2% (Figure 6c).

To evaluate the fluorescence detection threshold using the imprinted substrate, a calibration curve was generated (Figure 6d). Samples with various concentrations of MB were prepared and measured in both the nonpressed and pressed states. The detection threshold was calculated as^54^*I*_*threshold*_ = *I*_*background*_ + 3σ, and the corresponding concentration was calculated from the calibration curve. For the nonpressed state, the threshold was 0.88 μM, whereas the pressed state improved the threshold to 0.1 μM. These results highlight that the proposed piezoelectric system enhances detection sensitivity by nearly an order of magnitude.

## Conclusions

The piezoelectric properties of polymers, such as PVDF-HFP, can be utilized in the context of metal-enhanced fluorescence. The electric charges generated by the piezoelectric polymer film contribute to the amplification of the electric field at a metal-dielectric interface. Piezoelectric fluorescence enhancement was observed even on flat surfaces without distinct features, though it was more pronounced on imprinted surfaces with periodic geometrical features smaller than the excitation wavelength. The highest fluorescence amplification (∼85%) occurred at specific excitation wavelengths (532 nm), where the plasmon resonance was dominant. This effect is not limited to the specific geometry studied but is a general phenomenon that can be applied to various systems, with optimal results achieved when the excitation wavelength matches the plasmon resonance. Depending on the parameters of each system, the excitation wavelength that produces the highest enhancement will vary. Furthermore, for systems where distance is controlled, the enhancement factor due to piezoelectric charges may be even more significant compared to the values reported here. Finally, the effect can be probed even with sensitive samples, such as hybridized DNA assays, leading to the detection of lower concentrations.

## Methods

### Film Preparation

1.67g of the PVDF – HFP polymer was mixed with 7 mL of dimethylformamide and stirred on a hot plate at 30 °C for 16h to prepare the 20 wt % PVDF-HFP solution. Then the solution was poured on the silica nanopattern (imprinted sample) or on a coverslip (flat sample) and was spin-coated for 30 s at 1 V.

All films were subsequently thermally annealed on a hot plate at 90 °C for 1 h to promote solvent evaporation and polymer crystallization. After annealing, the imprinted films were carefully peeled from the template and affixed to clean glass coverslips using paper tape to ensure mechanical stability. The polymer concentration, spin-coating speed, and annealing temperature were selected to promote a high fraction of the β-phase in the PVDF-HFP film, based on conditions reported in the relevant literature.^31^ No significant variation was observed across different batches, and the desired nanostructure was consistently replicated at a satisfactory scale, in agreement with the literature.^55^ The β-phase content remained stable, with variations within ± 1%. The resulting film thickness is 5.5 μm. The samples were Ag-coated with an evaporation system (Sycon Instruments STM-100/MF thickness monitor, power 4 kW, deposition rate 0.8 nm/s) or Au-coated with EMITECH K575X sputter coater with a deposition rate of 0.2 nm/s. The thickness of metals is 10 and 15 nm correspondingly.

### Sample Preparation

200 μL of InP/ZnS quantum dots of 5 mg/mL in toluene concentration (Sigma – 776785) were diluted in 1000 μL of 50 mg/mL PMMA/toluene solution in a glass vial and then mixed. The QD concentration was 0.833 mg/mL, with a quantum yield of approximately 25%. A 70 μL aliquot of the resulting solution was drop-cast onto each sample and spin-coated for 30s at 0.5 V. Spin-coating the QDs in the PMMA solution ensured a random distribution, with some QDs positioned too close to the metal surface, leading to quenching, while others were at an ideal distance for fluorescence enhancement. The sample was left to dry in the air for 15 min and then was sealed by tapping a second glass slide on top. A second coverslip was placed over the QDs to enable mechanical deformation of the PVDF-HFP films when weight was applied. The PMMA layer and coverslip also protected the QDs from oxidation, preventing chemical degradation over time. This assembly creates a uniform interface for stress transfer and minimizes optical distortion. InP/ZnS QDs fluorescence emission spectrum peaks at 660 nm, consistent with the manufacturer’s data (∼650 nm), and has a full width at half-maximum (fwhm) of approximately 40 nm.

### Atomic Force Microscopy

The AFM images were obtained using a MFP-3D Asylum Research instrument operating in AC tapping mode. Monolithic silicon Tap300Al-G probes with aluminum reflective coating (BudgetSensors) were used to obtain the images. The specifications of the tip used for the image acquisition are 28 N/m force constant, ∼298 kHz resonance frequency (as measured from the instrument), and 125 μm (115–135 μm) length.

### Fourier Transform Infrared Spectroscopy

The ATR-IR measurements of nonsilver coated PVDF-HFP films were determined with the use of a Bruker, Alpha-Platinum ATR.

### UV/vis Reflection Spectroscopy

A LAMBDA 750 UV/vis/NIR spectrophotometer (PerkinElmer) was deployed to acquire the specular reflection spectrum of the Ag and Au-coated samples. An aluminum mirror was used to calibrate the instrument acting as a reference and the spectra were calculated as (*R*_*sample*_ – *R*_*background*_)/*R*_*background*_. The chosen laser slit was 2 nm.

### Surface Profilometry

Film thickness measurements were conducted using a Dektak 6 M stylus profilometer equipped with a 12.5 μm radius stylus. Scans were performed over a 2500 μm length with a resolution of 2.778 μm/sample and a duration of 3 s. A contact force of 2 mg was applied during measurement, and the system operated within a vertical measurement range of 65,500 nm.

### Fluorescence Spectroscopy

The fluorescence data were acquired with the use of a system, which comprises three monochromatic lasers power supplies (473 nm - CNIlaser MBL473 50 mW, 532 nm - CNIlaser MGL-III-532–200 mW, 633 nm - ThorLabs HRP120-1, 12 mW), an inverted optical microscope (IX71, Olympus), an Andor Kymera 328i spectrograph and an Andor iXon Ultra 897 EMCCD camera attached on it. The microscope’s filter wheel was equipped with beamsplitters and long-pass filters (Shamrock) for all excitation wavelengths.^56,57^ An ×20 objective (Olympus, UPLFLN 20×) was used to focus the laser on the sample. The movements of the sample were controlled through a high-speed, low-profile motorized xy scanning stage (MLS203-1, ThorLabs). Fluorescence measurements were performed with mechanical weights (calibrated stainless-steel weights) of various values (5, 10, 20, and 50 g) placed on top of the upper coverslip. To ensure consistent and evenly distributed mechanical loading, a clean glass coverslip was placed over the sample to create a flat, rigid surface for the weight. The weight was carefully centered by hand and adjusted under the optical microscope to ensure symmetric placement. The interface between the glass and the sample helped distribute the applied stress uniformly across the active area of the imprinted PVDF-HFP substrate (Supporting Information S19). Care was taken to avoid excessive force and to prevent laser reflections from the weights. To compensate for *z*-axis displacement due to weight application, the objective focus was adjusted for each frame. Fluorescence intensities were measured three times at 3–5 random spots for each weight to ensure repeatability and uniformity. Spectral reproducibility across spatial positions confirmed uniform load response. The samples were mounted on an automated xy stage with ± 0.001 mm precision, allowing consistent spot selection across all weight measurements.

### Simulation Details

**Figure 7:**
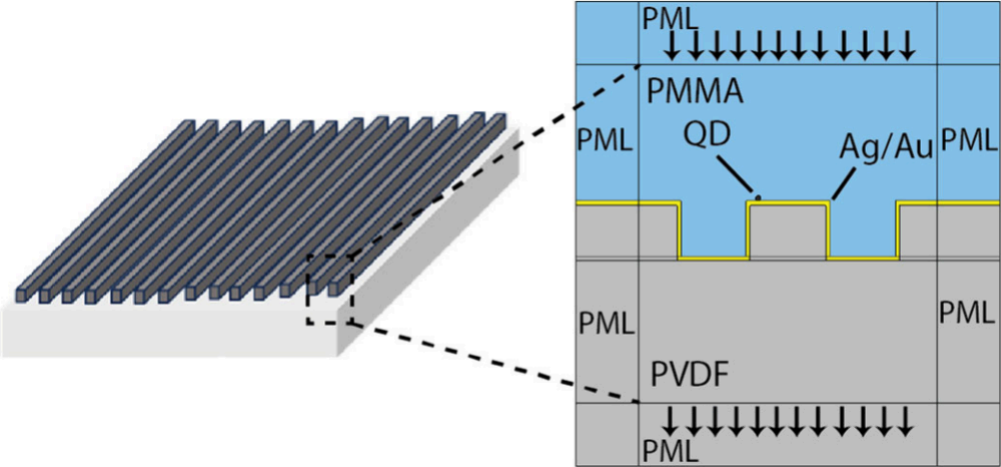
Geometry and material assignments of the finite element model.

Finite element analysis was carried out using the Electromagnetic Waves module in COMSOL Multiphysics. A 2D representation of two periodic unit cells of the imprinted surface was used to propagate the electromagnetic waves. (Figure 7) The refractive indices are taken as 1.4 + i0.02 for PVDF, and the refractive indices of Ag are taken from Johnson and Christy,^58^ the refractive indices of Au from Rosenblatt et al.,^59^ and the refractive indices of PMMA from Bodurov et al.^60^ An incident beam perpendicular to the sample’s surface and polarized to the *x*-axis is considered. Perfectly Matched Layers (PML) are used to surround the unit cell to prevent back-reflected waves. The enhancement factor is calculated from the electric field enhancement around the surface of the nanostructures:^34^
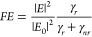
where *E* is the electric field that is locally enhanced on the quantum dot surface, *E*_0_ is the background electric field, and *γ*_*r*_ and *γ*_*r*_ are the radiative and thermal dissipated nonradiative powers. The electric field enhancement is calculated by integration of the local electric fields at the position of the quantum dot. For the calculations of the radiative and thermal dissipated nonradiative powers, an electric point dipole was placed in the center of the quantum dot. The surface integral of the time-averaged Poynting vector around the quantum dot gave the radiative losses, while the volume integral of the total power dissipation density on the nearby surface provided the thermal losses. To represent the generated piezoelectric potential from the application of a load, a surface current density is used on the surface. This effectively interacts with the propagation of the incident beam.

### Fluorescence Blinking

The blinking data was acquired with the setup for fluorescence spectroscopy. In this case, a x100 objective was used (Mitutoyo, M Plan APO 100x). An Andor iXon Ultra 897 EMCCD camera and a Kiralux 12.3 MP CMOS (ThorLabs, CS126MU) were used for data acquisition. A total of 31 quantum dots were recorded in both pressed and nonpressed states, with 25 dots imaged using a CMOS camera and 6 using a CCD camera. During acquisition, a bin time of 50 ms was used with the CMOS camera, while a 30 ms bin time was used with the CCD. For data processing, the time was set to start at 0.02 s, with each time range spanning 0.05 s (e.g., the first-time range is from 0.02 to 0.07 s). Events occurring within each time range were summed, and the average time was calculated for each range. The threshold between on and off events was chosen as *I*_*off_avg*_ + 1.1σ, where *I*_*off_avg*_ is the average intensity of off events. For the fit of occurrences over time, a simple power law was used *f*(*t*) = *ct*^–a^, within the linear space.

### Fluorescence-Lifetime Imaging Microscopy

A PicoQuant Microtime 200 fluorescence lifetime imaging confocal microscope is used to measure time-resolved photoluminescence. The samples were excited through a 40× objective (NA = 0.65) using picosecond laser pulses of 90 s duration at 405 nm with a repetition rate of 10 MHz and an integration time of 4 ms per pixel. The emission was collected back through the same objective. The PL decays were recorded over an 20 × 20 μm^2^. Spectral filtering is achieved using a combination of narrow-band and broadband emission filters. The emission is selected using a 500 nm filter with full-width-half-maximum of (100 ± 2) nm. The PL lifetimes were measured at room temperature. For the fitting of the decay curves the following equation was used: . The average lifetime was calculated as follows: .

### DNA Preparation

A 10 μΜ stock solution of Probe DNA (5′-TGT GTT TAC GAG CGG TTT CG/3ThioMC3-D/-3′) was prepared by adding 500 μL of Phosphate-Buffered Saline (PBS) to an Eppendorf tube containing the probe ssDNA. This step was repeated with an Eppendorf containing fully complementary, Methylene Blue labeled target DNA (ssDNA 5′-TGT GTT TAC GAG CGG TTT CG/3ThioMC3-D/-3′) to make a 10 μM concentration.

### Probe Immobilization

100 μL of Probe ssDNA, 10 μL of Mercaptocyclohexanol (MCH), and 890 μL of PBS were added to an Eppendorf tube and spun. The solution was dropped onto the Au-PVDF polymer to cover the surface and left for 16 h. After incubation, the surface was washed with PBS buffer to remove unbound probes, followed by drying using nitrogen gas.

### Target DNA Hybridization

A 10 μM solution of target Methylene Blue labeled DNA was applied to the top of the Au-PVDF surface. The sample was then set on a hot plate at 90 °C for 2 min and then cooled for 1 h to induce hybridization. After this step, the surface was washed with PBS buffer and dried with nitrogen to prepare it for measurement.

### Control Experiment for Nonspecific Binding

A nonspecific binding control was conducted to ensure the specificity of the DNA hybridization process. In this experiment, a single-stranded Probe Gap A DNA was attached to the Au-PVDF surface, subsequently introducing a noncomplementary Shigella target DNA labeled with Methylene Blue (5′-CCT TTT CCG CGT TCC TTG A-3′). After incubation under similar conditions as the specific hybridization experiment, the material was rinsed with PBS buffer and dried with nitrogen before measurement. This control was performed to assess nonspecific interactions and ensure that any observed signal in the fluorescence measurements arises from specific probe-target hybridization rather than unintended adsorption or binding (Supporting Information Figure S15).

## Supporting Information Available

Reflectance data for Ag and Au coating samples; AFM images, SEM images and surface profile for imprinted and linear substrates; voltage control measurements; fluorescence spectroscopy measurements for Au-coated imprinted samples and Ag-coated linear samples; simulation results for Au-coated samples; metal thickness simulations versus electric field enhancement; methylene blue absorption spectrum; methylene blue emission spectra and concentration graph up to high concentrations; schematic of piezoelectric enhanced fluorescence process; weight distribution factor (PDF)
