# Supplementary material for: Piezoelectric-Driven Amplification of Plasmon-Enhanced Fluorescence for Advanced Sensing Applications
Source: ACS Appl Mater Interfaces. 2025 May 5;17(19):28881–93. doi: 10.1021/acsami.5c03428 (PMC12086765; doi:10.1021/acsami.5c03428)
Supplement: Supplementary file 1 — am5c03428_si_001.pdf [file am5c03428_si_001.pdf]

Supporting information

Piezoelectric-Driven Amplification of Plasmon-enhanced Fluorescence for Advanced Sensing Applications

Eni Kume[1*], Ghadeer Almohammadi[2,3], Dominik Duleba[2], Aeshah Farhan M Alotaibi[1,4], Rongcheng Gan[1], Kseniia Mamaeva[5,6], A. Louise Bradley[5,6], Robert P. Johnson[2] & James H. Rice[1*]

(1) School of Physics, University College Dublin, Belfield, Dublin 4, D04 V1W8, Ireland

(2) School of Chemistry, University College Dublin, Belfield, Dublin 4, D04 V1W8, Ireland

(3) Chemistry Department, College of Science, University of Hafar Al Batin, Hafar Al-Batin, 31991, Saudi Arabia

(4) Department of Physics, College of Science and Humanities, Shaqra University, Shaqra, 11961, Kingdom of Saudi Arabia

(5) School of Physics and AMBER, Trinity College Dublin, Dublin 2, D02 PN40, Ireland

(6) IPIC, Tyndall National Institute, Cork, T12 R5CP, Ireland

* Email: eni.kume@ucd.ie; james.rice@ucd.ie

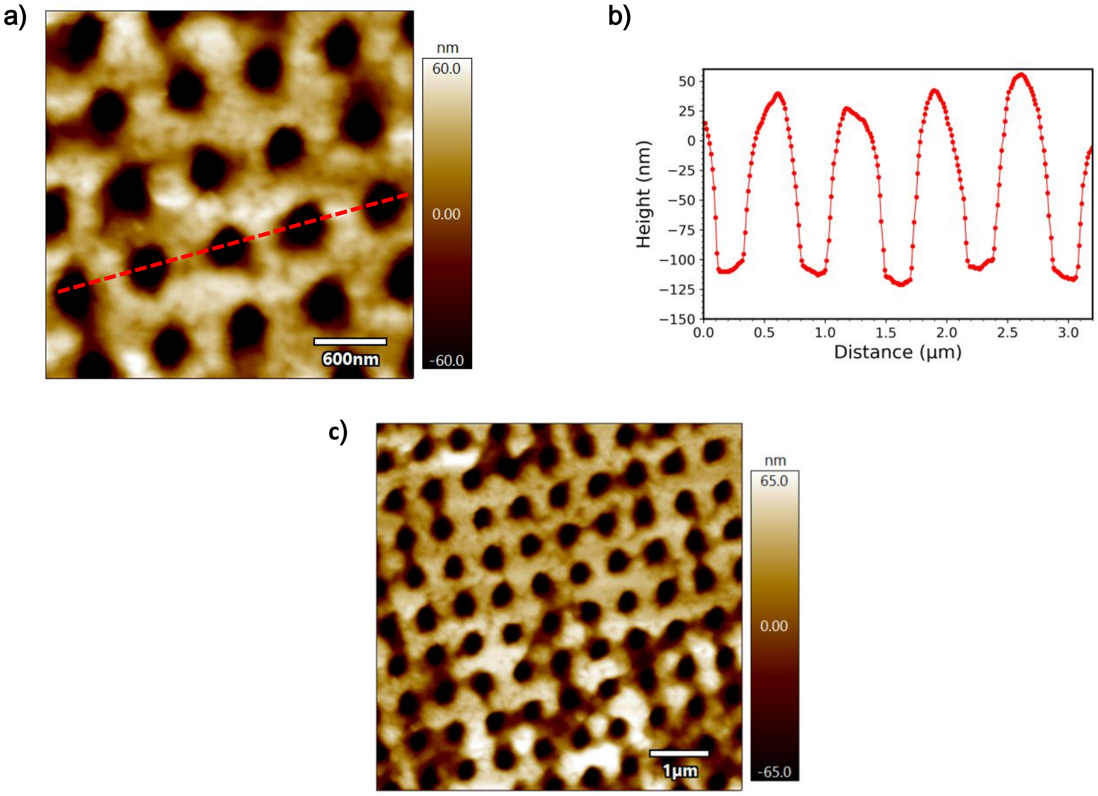

Figure S1: ***a)*** *AFM image of the imprinted pattern. Dotted line corresponds to the surface profile.* ***b)*** *Surface profile of (a).* ***c)*** *Zoomed out AFM image of the imprinted (holes) sample.*

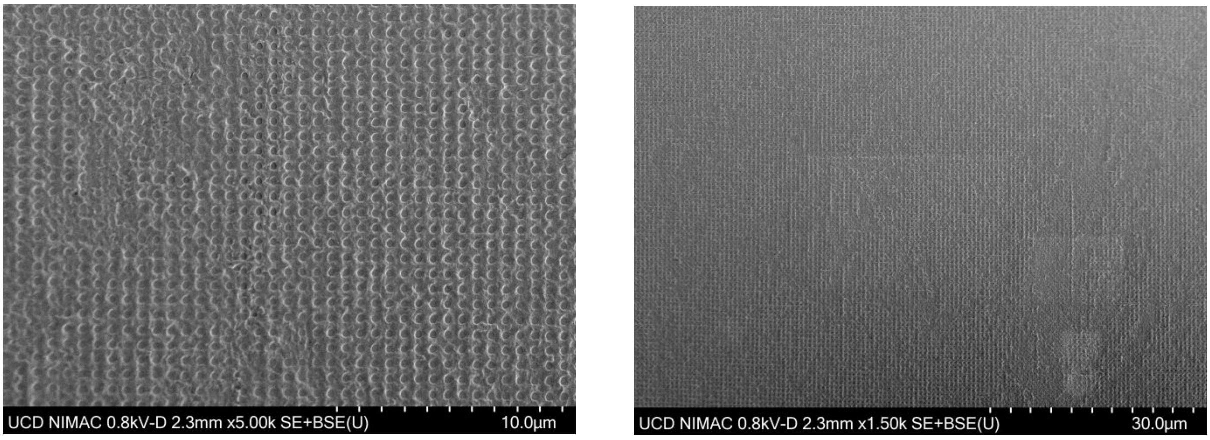

Figure S2: *Large-area SEM images of the imprinted pattern.*

a)

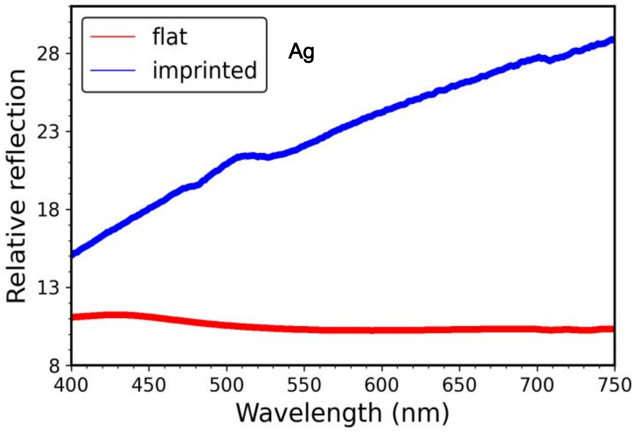

Figure S3: *Reflection spectra of silver-coated flat and imprinted films.*

b)

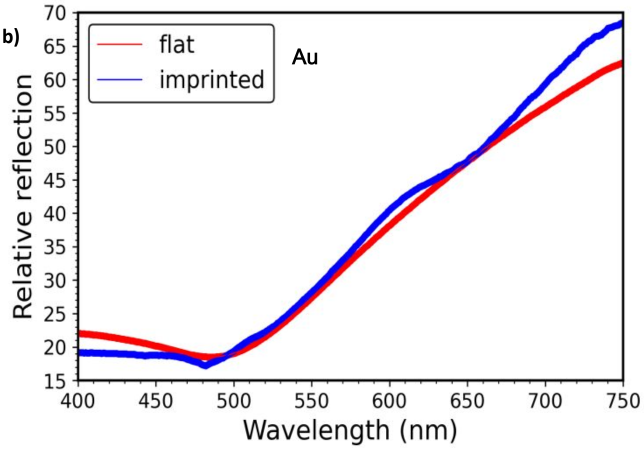

Figure S4: *Reflection spectra of gold-coated flat and imprinted films.*

a)

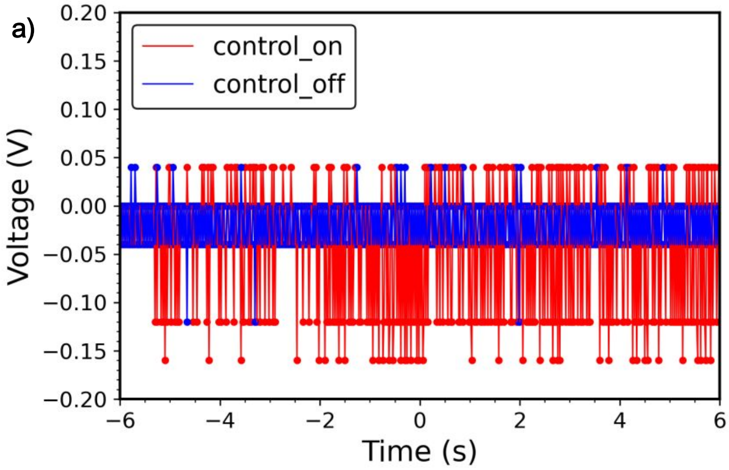

b)

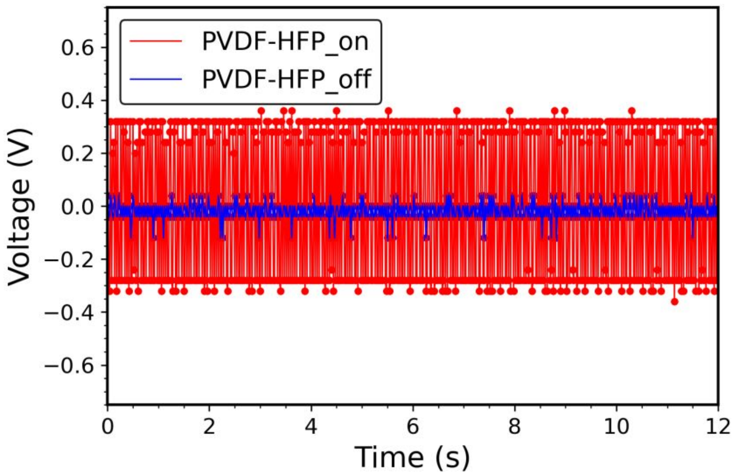

*F**igure S5:** Voltage over time measurement during a 220Hz acoustic sin wave excitation of **a)** control sample **b)** PVDF-HFP flat film.*

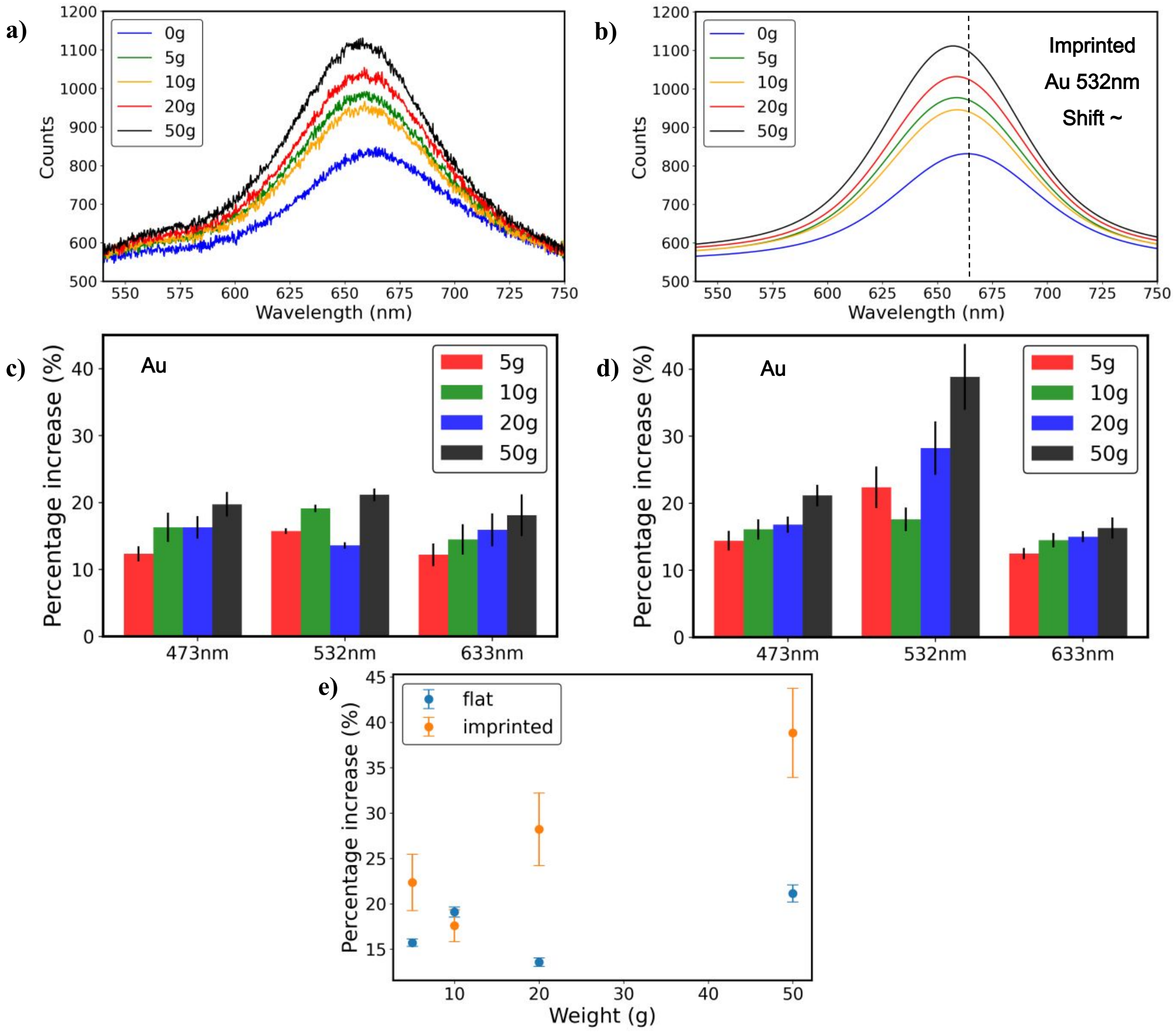

***Figure S6: a)** Average emission spectra of InP/ZnS QDs in PMMA/toluene solution, spin-coated on a gold-coated imprinted (hole features) PVDF–HFP film under different mechanical weights, with 1 mW, 532 nm laser power.* ***b)** Fitted gaussian curve of the average emission spectra.* ***c-d)** Fluorescence percentage enhancement of InP/ZnS QDs on Au-coated PVDF–HFP samples for various applied weights and excitation wavelengths.* ***e)** Scatter plot showing the fluorescence percentage increase of the tested samples under different weights for 532nm excitation wavelength.*

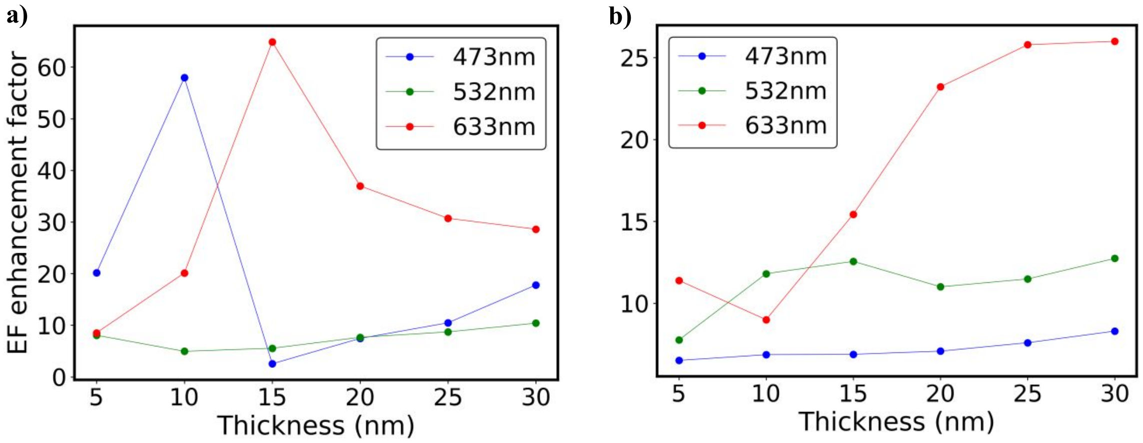

Figure S7: *COMSOL simulated electric field enhancement factor as a function of the metal thickness for three different excitation wavelengths.* ***a)*** *Silver,* ***b)*** *Gold.*

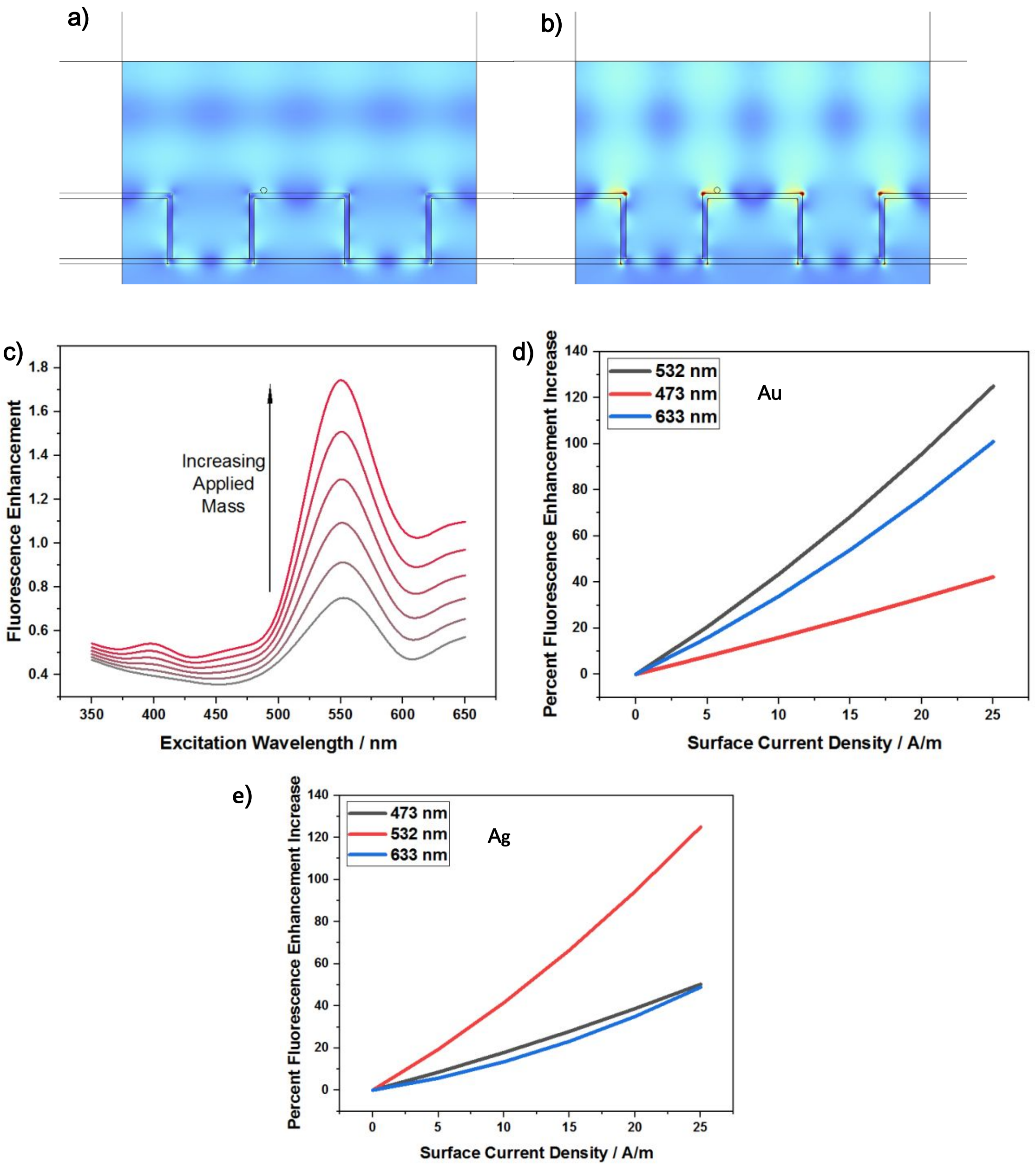

Figure S8: *2D geometry of the imprinted gold-coated sample used for simulation calculations:* ***a)*** *Electric field enhancement compared to a flat gold-coated geometry before pressing at a 532 nm excitation wavelength.* ***b)*** *Electric field enhancement compared to a flat geometry after pressing the PVDF-HFP substrate at a 532 nm excitation wavelength.* ***c)*** *Simulated fluorescence enhancement of InP/ZnS quantum dots on gold-coated imprinted PVDF-HFP as a function of excitation wavelength, compared to a flat silver-coated substrate. The grey region corresponds to no applied weight.* ***d)*** *Simulated percentage fluorescence enhancement for the excitation wavelengths used in experiments, as a function of applied weight for gold-coated samples.* ***e)*** *Simulated percentage fluorescence enhancement for the excitation wavelengths used in experiments, as a function of applied weight for silver-coated samples.*

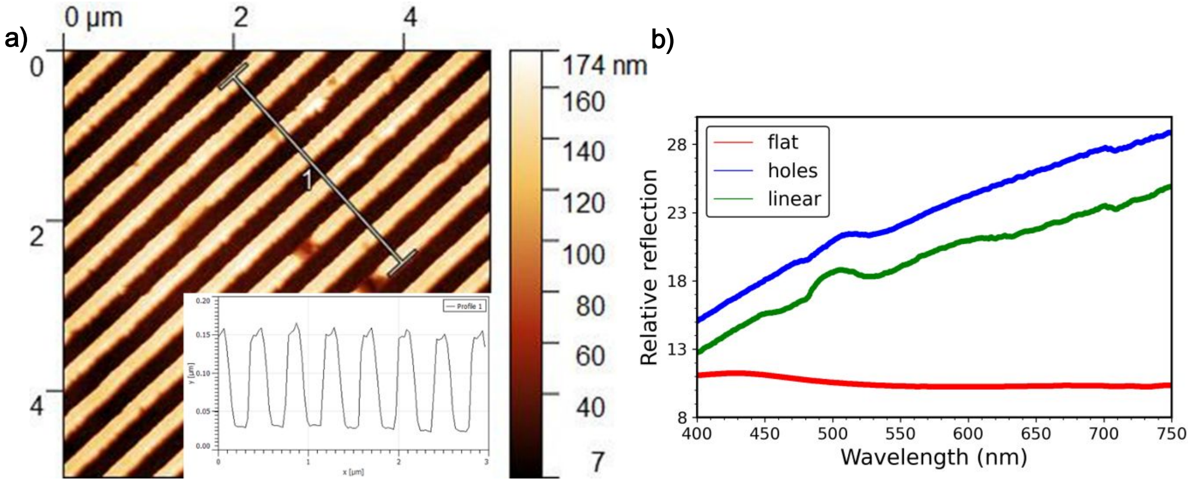

Figure S9: ***a)*** *AFM image of the imprinted* ***linear*** *pattern. Insert: Surface profile of the imprinted pattern.* ***b)*** *Reflection spectra of silver-coated flat and imprinted films*

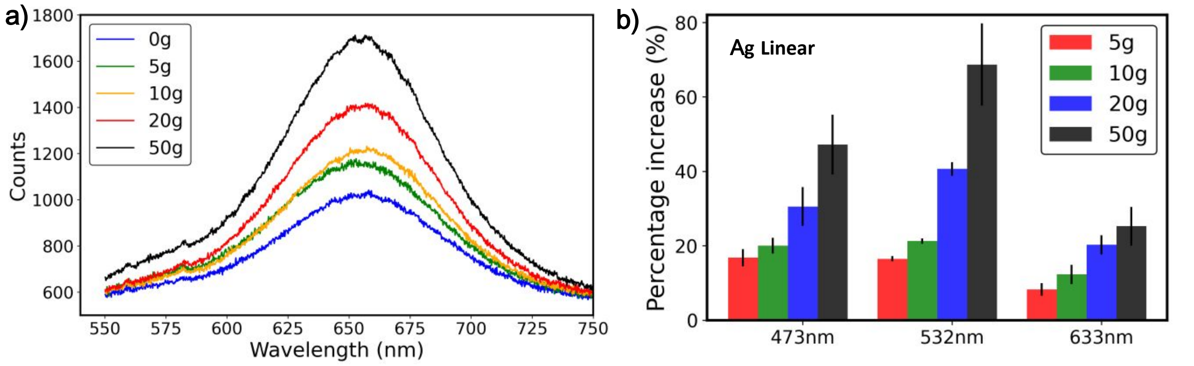

Figure S10: ***a)*** *Average emission spectra of InP/ZnS QDs in PMMA/toluene solution, spin-coated on a silver-coated imprinted (****linear features****) PVDF–HFP film under different mechanical weights, with 1 mW, 532 nm laser power****.*** ***b)*** *Fluorescence percentage enhancement of InP/ZnS QDs on Ag-coated linear PVDF–HFP samples for various applied weights and excitation wavelengths.*

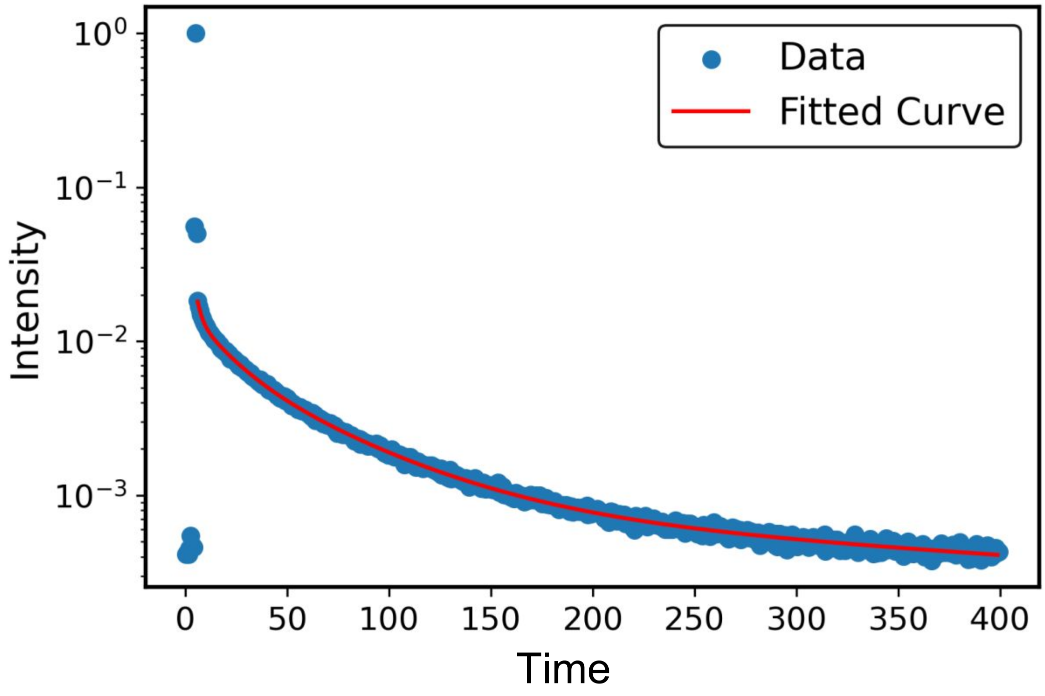

Figure S11: *4-exponential fit of lifetime measurements on glass substrate.*

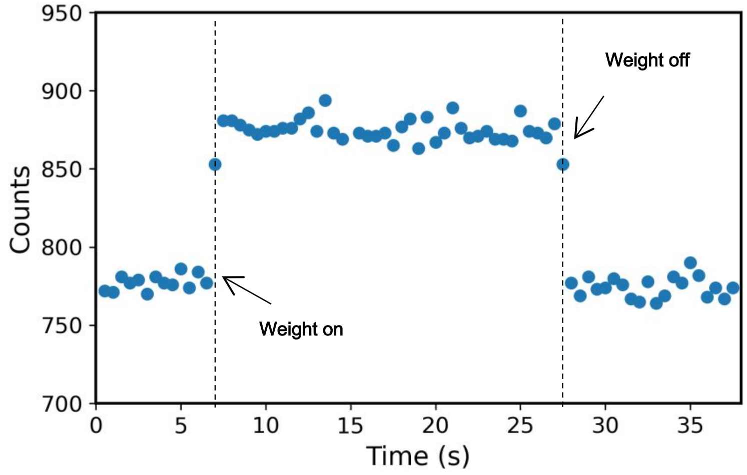

Figure S12: *Another sequential fluorescence measurements of InP/ZnS QDs on Ag-coated imprinted PVDF–HFP films were performed while adding/removing a 5 g weight. Each point represents the maximum intensity (in counts) of the fluorescence spectrum for a 0.5 s camera exposure time.*

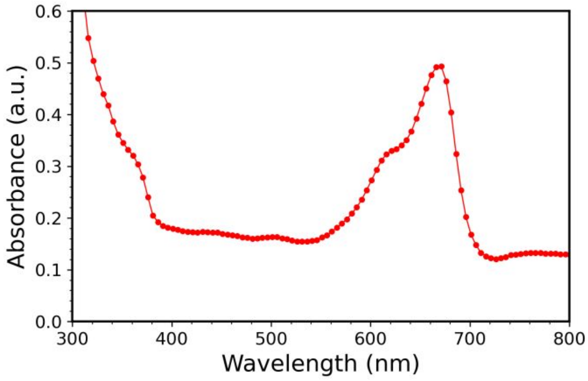

Figure S13: *Absorption spectrum of methylene blue.*

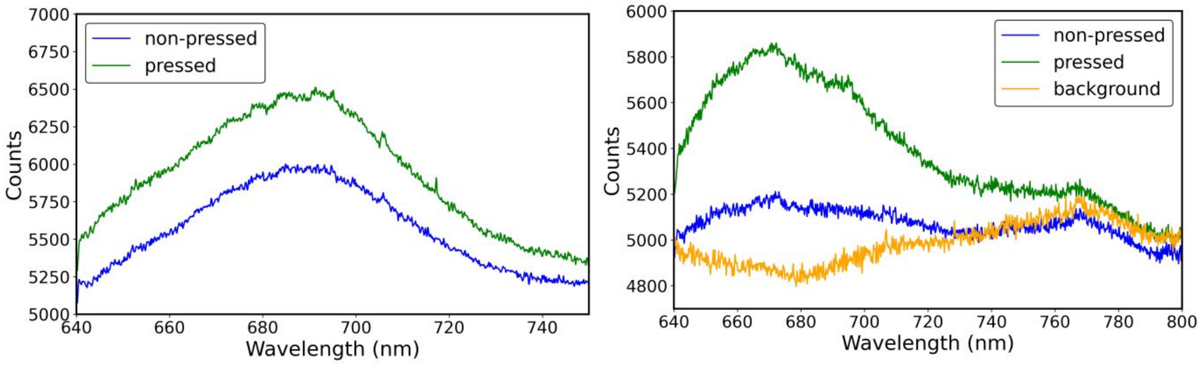

Figure S14: *Examples of fluorescence signal of MB labeled DNA assay imprinted sample (8µM, left and 3µM, right) before and after pressing.*

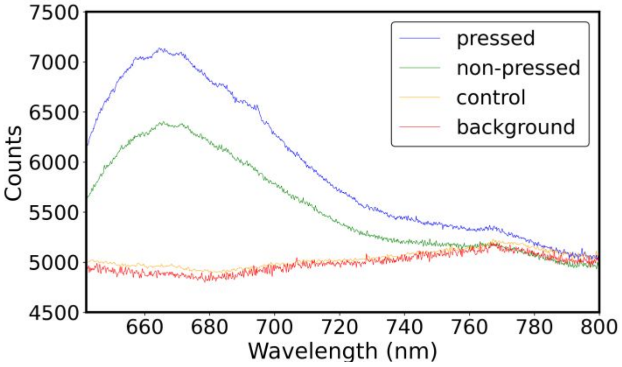

Figure S15: *Fluorescence signal of MB labeled DNA assay flat sample (10µM,) before and after pressing. Control refers to non-specific binding control sample (10µM) to assess non-specific interactions.*

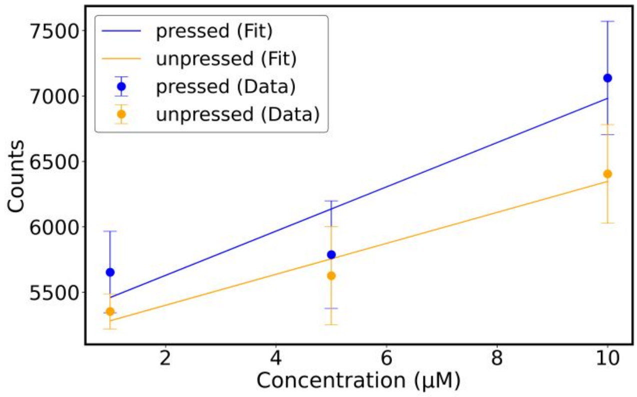

Figure S16: *Evolution of fluorescence intensity of MB at varying concentrations for non-pressed and pressed imprinted samples. Linear region of detection from 0.1µM to 10µM. For higher concentrations saturation occurs, with complete collapse at high concentrations (1000µM).*

	**Slope**	**Intercept**	R^2
**Non-pressed**	225.0	4921.8	0.883
**Pressed**	346.7	5081.2	0.968

Table S1: *Values of linear fit for fluorescence intensity of MB at varying concentrations for non-pressed and pressed imprinted samples.*

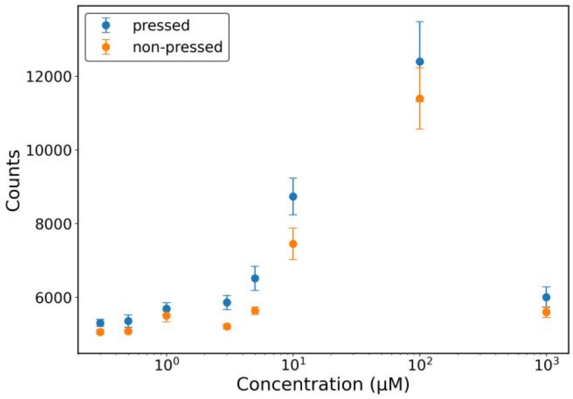

Figure S17: *Evolution of fluorescence intensity of MB at varying concentrations for non-pressed and pressed flat samples.*

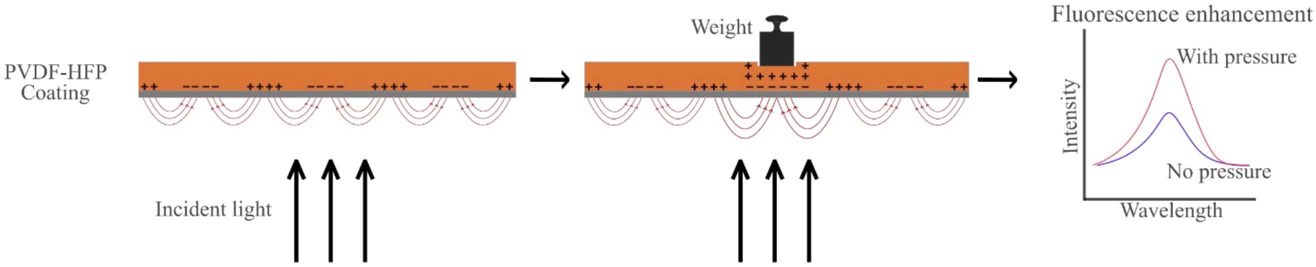

***Figure S18:** Simplified schematic of the piezoelectric enhanced fluorescence.*

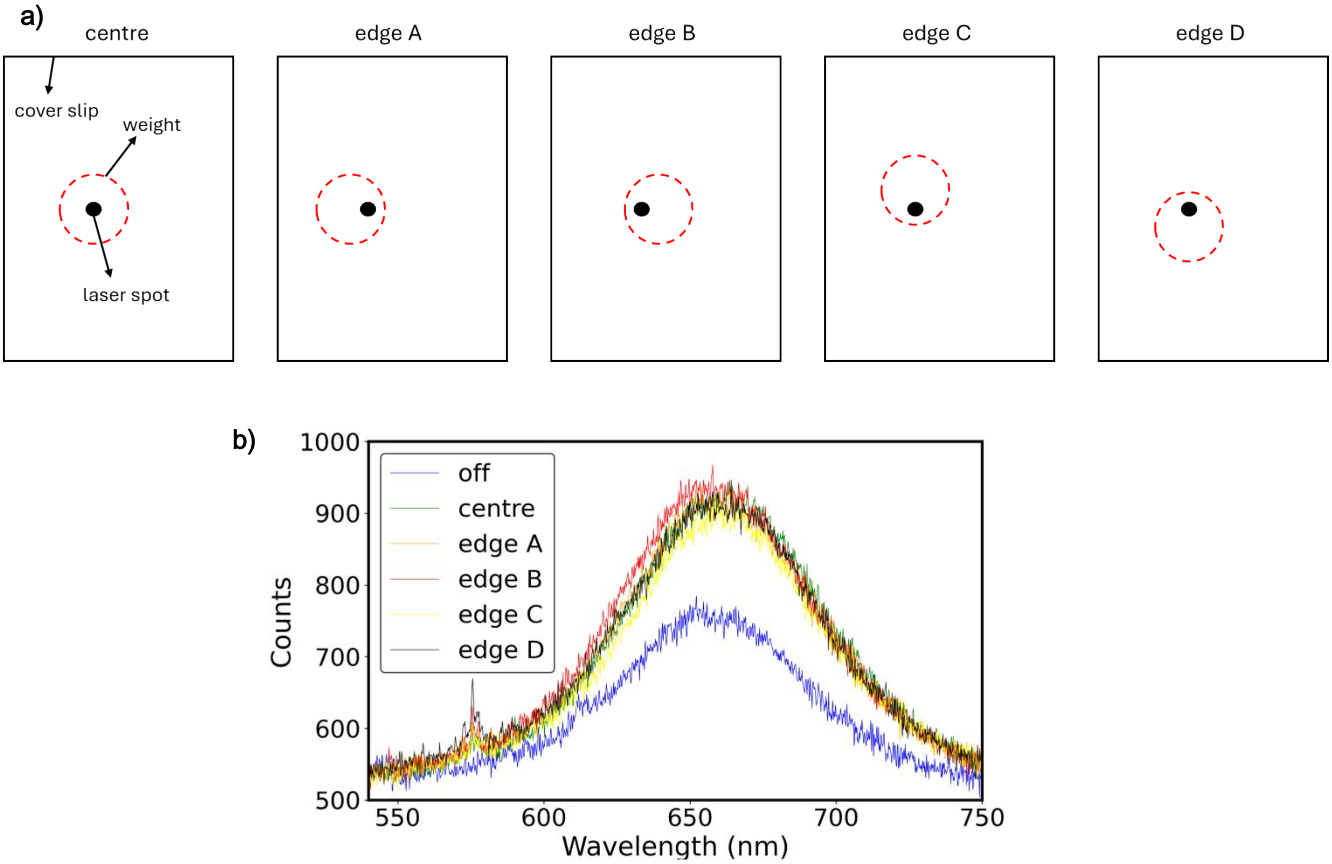

Figure S19: a) Schematic illustrating the position of the weight relative to the laser spot. **b)** Fluorescence signal of InP/ZnS quantum dots (QDs) at various weight positions.
